# Supplementary material for: Benchmarking of alignment-free sequence comparison methods
Source: Genome Biol. 2019 Jul 25;20:144. doi: 10.1186/s13059-019-1755-7 (PMC6659240; doi:10.1186/s13059-019-1755-7)
Supplement: Supplementary file 3 — Review history. (DOCX 32 kb) [file 13059_2019_1755_MOESM3_ESM.docx]

Review History

**Comments to author and authors’ point-by-point responses to the reviewers’ comments:**

**Reviewer 1**

In the manuscript, Zielezinski et al. set to systematically compare methods which use alignment-free approaches to compare nucleotide or amino-acid sequences. For that they built a web-based infrastructure that uses a set of pre-defined datasets and comparison/evaluation tactics. The datasets used are of 5 types which are usually subjected to sequence comparisons. They then used their infrastructure to compare 24 softwares, which sums to 74 methods when using the softwares with different set of parameters. They then explain in detail the difference and best practice for each of the 5 data-types. The web tool built, is targeted to be the center of comparison between sequence comparison methods, for both developers and users, as new methods information can be easily incorporated with the curated data present in the manuscript. Lastly, although the focus is solely on alignment-free approaches the web tool can be used also with alignment-based methods.

The manuscript is well written and create a bench-mark and order for users and developers in this area of research. The many software and parameter set used create a very comprehensive and useful resource.

I have a few minor comments:

1. I think a bit more background for the non-specialized user, both in the introduction and also in the different section can improve the manuscript. The broad-readership of this journal might not be fully familiar with alignment-free methods, and I think with minor changes this can be a much more approachable article. For example, the nicely written summary on each method in the supplementary is very useful for a newbie to this field.

*Response: We very much appreciate this important suggestion as the main intention of our paper was to describe the performance of alignment-free tools not only to software developers but also to non-specialized users of these tools. We agree with the Reviewer that the broad-readership of the Genome Biology journal might not be fully familiar with the underlying concepts of alignment-free sequence comparison. As the description of these concepts would require a considerable amount of manuscript space to be fully understandable by a broad audience, and may not be as interesting as are practical aspects of the tools, we now point the reader to review articles providing in-depth explanation of alignment-free approaches to sequence comparison (page 4).*

*In addition to review papers referenced in the Introduction, we provide a brief description of each of 24 alignment-free tools in Methods, under the “Alignment-free tools” section. This summary describes the main ideas behind those tools rather than technical details, and it was prepared and reviewed by the developers of those tools.*

2. In the introduction it is written "do not scale with the very large data sets that are available today" - please provide a reference or further explain.

*Response: We have provided the references* [*(Bernard et al. 2019; Chan and Ragan 2013)*](https://paperpile.com/c/jcDzMT/xTAh+oQAA) *to support the statement about the limited scalability of multiple sequence alignment (page 3).*

3. Proteins databases (e.g. family / class) - it would be helpful to give an explanation to what each of this level correspond to.

*Response: Thank you for this valuable comment. In the Methods section (page 21), we have added a short explanation for each of the SCOP levels (i.e., class, fold, superfamily and family).*

4. nRF measure - it would be helpful to give a short explanation to how this is calculated, given the broad use of it in the manuscript.

*Response: Thank you for this helpful suggestion. In the Methods section (page 32), we have explained, in simple terms, how the nRF distance is calculated.*

5. In the discussion: "...The differences observed were due to the different methods of sequence data preprocessing applied by the two tools — alfpy projects sequences into a vector of k-mer frequencies, whereas AFKS represents sequences as k-mer count vectors with the inclusion of pseudocounts. This sequence data preprocessing in alfpy and AFKS has the highest impact on the performance of methods.." - This difference in pre-processing and why it matters was not clear.

*Response:AFKS and alfpy calculate dissimilarity between two sequences based on k-mer occurrences within the sequences being compared. Both tools use the exact same dissimilarity measures to compare k-mer occurrences such as Euclidean distance, Manhattan distance and Canberra distance.*

*It was thus surprising that running AFKS and alfpy with the same input sequences, the same k-mer size (e.g., k = 2), and the same dissimilarity measure (e.g., Canberra distance) produces significantly different results. The prime example is the CRM data set, where AFKS--Canberra obtained a performance score of 54, while alfpy--Canberra had a performance score of 74, which is the highest performance score among all the tools that we evaluated.*

*The difference in performance between AFKS and alfpy is due to the different ways to represent k-mer occurrences in the sequences. AFKS represents sequences as k-mer counts (i.e., counts how many times each particular k-mer is present in a sequence) and alfpy uses k-mer frequencies (i.e., a relative abundance of each particular k-mer obtained by dividing its count by the sum of all counts). In addition, AFKS adds 1 (pseudo-count) to each of the k-mer counts to prevent division by 0 as some dissimilarity measures implemented in AFKS require dividing by k-mer occurrences.*

*The sequence data preprocessing in AFKS and alfpy makes an interesting case — in our opinion — that different implementations of the same alignment-free algorithm, run with the same input parameter values, can deliver different results.*

**Reviewer 2**

The authors describe a method for assessing the performance of multiple sequence comparison algorithms, specifically those that do not use multiple-sequence-alignment. They identify five sequence comparison use cases that are equivalent to finding similarity or dissimilarity scores between all pairs of sequences being analysed. For each of these use cases they generate a trusted truth set and assessment criteria, and evaluate multiple tools at the task. Further, they provide a website to publish the results and upload new results as tools are developed.

In the introduction they describe the limitations and shortcomings of multiple-sequence-alignment based comparisons.
In the results section they describe the benchmarking website as well as the five tasks at which tools are benchmarked (Gene tree inference, regulatory element analysis, genome-based phylogeny from assembled genomes and raw sequencing reads, and horizontal gene transfer).
In the methods sections they describe in detail how they benchmarked the tools at each task. They include a comprehensive review of the tools used for alignment-free sequence comparison.

The benchmarking is thorough and the visualisations implemented on the website are impressive and useful.

Suggested changes are as follows:

For horizontal gene transfer: Please provide motivation for choosing 0, 250, 500, 750 and 1000 for "HGT events attempted in the set at each iteration of the simulation process of genome evolution"

*Response: Our approach for simulating HGT in genome sequences follows the approach adopted in an earlier study* [*(Bernard, Chan, and Ragan 2016)*](https://paperpile.com/c/jcDzMT/1KQI)*. This simulation strategy has been shown in that study to yield data that are appropriate to assess the performance accuracy of a range of AF methods in recovering the reference phylogeny (i.e. the phylogeny with zero simulated HGT events). Bernard et al. (2016) was cited in the Methods section where we describe the strategy in detail, and we now justify our strategy by citing this reference in the Results section (page 16).*

I don't understand the sentence: "Additionally, no statistical substitution models are currently available for assessing the evolution of complete genomes". From large resequencing projects and sequencing of related species, we have a good idea about the patterns of variation seen across the human genome and where e.g. conserved regions are. Perhaps I am misunderstanding this line.

*Response:* *We agree the sentence might not have been clear without further explanation. Most whole-genome aligners use models or scoring schemes that assume homogeneous rates of evolution across the genome* [*(Dewey 2012)*](https://paperpile.com/c/jcDzMT/lcYt)*. This assumption is obviously violated in real data as genomes undergo both local (i.e., including small number of nucleotides) and large-scale mutational processes (i.e., gain/loss/duplication of large segments) at various rates. In addition to substitution models, genome aligners need to model genome rearrangements, such as inversions, translocations, chromosome fusions, chromosome fissions, and reciprocal translocations as well as unbalanced rearrangements that lead to copy number change, such as tandem and segmental duplications* [*(Earl et al. 2014)*](https://paperpile.com/c/jcDzMT/9Jhs)*.*

*We have removed from the manuscript the phrase “Additionally, no statistical substitution models are currently available for assessing the evolution of complete genomes” and extended the previous sentence to inform the reader about potential problems with whole genome alignment (page 12).*

In the Results section 'Raw sequencing reads':

- It should be made clear that results are for simulated reads, not real sequencing data (if I'm reading the Methods section correctly).

*Response:* *We apologize for this oversight. In the first sentence of the “Raw sequencing reads” section (page 14), we have clarified that raw sequencing reads are simulated.*

It's not obvious that real data couldn't be used for this evaluation. Real sequencing data may have a different error modes, chimeric reads from library prep, contamination etc. It would be good to know how well these approaches hold up with real data.

*Response:* *We agree, in principle it would be possible to use real-world sequencing reads in addition to the simulated reads that we used. However, this would involve a number of difficulties: we do not exactly know a-priori how many and which errors we can expect in real reads, we don’t know if or how many chimeric reads there are etc. So it would be difficult to tell to what extent errors in the produced trees would be due to errors in the reads, or due to flaws of the evaluated software. In any case, it would be necessary to involve additional experts as co-authors, to find suitable data sets, and to discuss the above issues in detail. We’re afraid, all this would be far beyond the scope of the present paper. We think it would be very interesting to carry out a dedicated study based on a broad range of real-world reads, but not as part of the present paper.*

The first sentence should reference Table 2, not Table 1.

*Response: Thank you for spotting this unfortunate misnumbering of Tables. The correct label has been provided for the table in the sentence (page 14).*

A benchmark of programs should record the computational resources required for each result, such as wall clock time, peak memory and CPU time. Submitted data on the website might be hard to compare, but for the comparisons run in the paper these metrics would be useful.

*Response:We thank the Reviewer for bringing this very important point. As the Reviewer said, new alignment-free methods submitted to the benchmarking service are infeasible to compare in terms of computational time and memory usage since these tools are run on the submitters’ machines. At first sight, execution runtime would appear to be measurable objectively for the 24 alignment-free tools included in this paper. However, we had to refrain from reporting it in the paper for the following reasons.*

*First, half of the tested AF tools were run by their developers on different machines. To unify these tool runs, we were initially inviting participants to share results together with their software in a Docker container following the bioboxes standard* [*(Belmann et al. 2015)*](https://paperpile.com/c/jcDzMT/B7xT)*. However, the participating packages exhibit some degree of dependence upon hardware specifications (e.g., the phylonium tool is significantly faster on CPUs that support AVX2 than machines with only SSE2), which prevented us from running every software by ourselves.*

*Second, we attempted to use a proxy approach* [*(Sage et al. 2015)*](https://paperpile.com/c/jcDzMT/xQEp)*, which determines the computational runtime by analyzing the answers received from the participants and by normalizing it by the power of their machine. However, in the course of our project many alignment-free tools that we tested underwent significant improvements in terms of speed and memory usage (e.g., in just five months the Skmer tool has been upgraded from version 2.0.0 via 2.0.1 and 2.0.2 to 3.0.0).*

*Finally, since there does not seem to be a way of reporting run-time and memory usage on our web service, the information provided in the paper will soon be outdated and misleading. Therefore, following similar community-based benchmarking services — e.g., Quest for Orthologs (QfO)* [*(Altenhoff et al. 2016)*](https://paperpile.com/c/jcDzMT/t78M)*, Critical Assessment of Metagenomic Interpretation (CAMI)* [*(Sczyrba et al. 2017)*](https://paperpile.com/c/jcDzMT/GsuQ) *— we decided not to record the computational time and memory usage of the tools we tested.*

In the simulation of horizontal genes (Methods, p23), it would be useful to understand the resulting distribution of similarities between the genes simulated. Is this a diverse or conserved set of genes?

*Response: The HGT events were simulated to occur at random, i.e. anywhere along a genomic sequence and between any pair of genomes in a set. The sequence similarity of the horizontally transferred genes and the receptivity of an HGT event are determined by other parameters in EvolSimulator, including mutation rate per iteration of simulation, and the divergence factor that prevents horizontal transfer of highly divergent genes (as we expect HGT implicating these genes to rarely occur); we provide these details in the Methods section (page 23). The overall simulation strategy for HGT was adopted from Bernard et al. (2016); see also our response above. Per the Reviewer’s suggestion, we have supplemented the manuscript text (page 16) with one sentence explaining the HGT data simulation procedure.*

The authors don't provide a commentary on why some algorithms perform better than others. Some discussion would be helpful for researchers who need to pick or develop one of these tools to solve similar problems.

*Response: All the tools are a combination of sequence preprocessing and distance calculating algorithms. In some cases (see our responses to Reviewer 1) the same distance measure produces distinct results. In this case it was easy to pinpoint the difference in performance to preprocessing step. We do believe that this step may be crucial in performance of other tested tools, but we can not be absolutely sure without exploring the programming code of each of the tools. Therefore, we refrain ourselves from making any solid conclusions on why some algorithms perform better than others, limiting only to one example in the discussion.*

I am interested if the authors could propose reasons for all tools performing badly on the transmembrane protein families Bambi and Asterix.

*Response: We were asking ourselves the very same question on several occasions during this project. The question is very interesting as both the transmembrane protein families do not stray from other protein families used in the benchmark. For example, in comparison to other protein families, Bambi and Asterix can be characterized by (i) medium number of protein members (42 and 39, respectively), (ii) small number of duplication events, and (iii) presence of single protein domain only. Also, the average sequence identity among the family members of Bambi and Asterix is not the lowest in contrast to the remaining protein families (60% and 37%, respectively).*

*Table 1. Features of protein families obtained from SwissTree.*

| *Protein family* | *No. members* | *No. duplications* | *No. domains* | *Average sequence identity** | *Standard deviation sequence identity* |
| --- | --- | --- | --- | --- | --- |
| *Popeye* | *49* | *2* | *1* | *36%* | *17%* |
| *NOX (NADPH oxidases)* | *54* | *10* | *3-4* | *40%* | *17%* |
| *V-type ATPase beta subunit* | *49* | *5* | *3* | *74%* | *9%* |
| *Serine incorporator* | *115* | *17* | *1* | *32%* | *13%* |
| *SUMF* | *29* | *1* | *1* | *46%* | *13%* |
| *Ribosomal protein S10/S20* | *60* | *5* | *1* | *32%* | *19%* |
| *Bambi* | *42* | *2* | *1* | *60%* | *32%* |
| *Asterix* | *39* | *2* | *1* | *37%* | *25%* |
| *Cited* | *34* | *6* | *1* | *36%* | *20%* |
| *Glycosyl hydrolase 14* | *159* | *39* | *1-2* | *35%* | *12%* |
| *Ant transformer* | *21* | *7* | *2* | *42%* | *17%* |

** Average sequence identity among protein members was calculated as an average of global sequence alignments between every pair of members within a protein family.*

*However, Bambi and Asterix protein families show the highest variation in sequence identity (±32% and ±25%, respectively), meaning that these families are mixtures of protein sequences with very high and very low sequence identity. Such heterogeneity in sequence identity may be the reason why all alignment-free tools performed badly. For example, lower k-mer sizes will successfully classify most of low-sequence-identity relations and misclassify many high-sequence-identity relations as opposed to greater k-mer size that will successfully classify most high-sequence-identity relationships, but misclassify most of low-sequence-identity relations. Whatever the k, k-mers will be too short to be specific enough in highly conserved proteins and too long to be sensitive enough in protein sequences with little identity.*

*Notably, the standard alignment-based approach is also the least accurate in the case of Bambi and Asterix protein families.*

*Table 2. The normalized Robinson-Foulds (nRF) distance for neighbor-joining trees inferred using ClustalW alignments with default parameters.*

| *Protein family* | *nRF* |
| --- | --- |
| *Popeye* | *0.21* |
| *NOX (NADPH oxidases)* | *0.18* |
| *V-type ATPase beta subunit* | *0.36* |
| *Serine incorporator* | *0.16* |
| *SUMF* | *0.12* |
| *Ribosomal protein S10/S20* | *0.31* |
| *Bambi* | *0.59* |
| *Asterix* | *0.58* |
| *Cited* | *0.25* |
| *Glycosyl hydrolase 14* | *0.23* |
| *Ant transformer* | *0.30* |

Given the reliance on the normalized Robinson-Foulds metric, a couple of sentences on its usefulness and limitations would be helpful for the reader. You may wish to refer to a recent review of tree comparison metrics, e.g.: Practical Performance of Tree Comparison Metrics; Kuhner, Yamato, Systematic Biology (2015) <https://doi.org/10.1093/sysbio/syu085>

*Response:Following the Reviewer’s suggestion, we have added a paragraph in the Methods section (page 32) concerning the calculation, usage and limitations of the normalized Robinson-Foulds metric. Thank you for providing the reference - we have included it in the manuscript.*

p6: First sentence in section "Alignment-free method catalog": "data" should be "data set".

*Response:Thank you. The word “data” has been changed to “data set” (page 6).*

p8: Under "Protein sequence classification": The phrase "56 tool-measure variants" is confusing - I prefer the phrase "tool variant" that you've used elsewhere.

*Response:We agree; we made sure to use “tool variant” consistently throughout the manuscript.*

p33: Should read '100' not '1' in: This way, the best-performing method in a given data set receives a score of 1,

*Response:Thank you for spotting this typo. We have corrected it (page 34).*

Tables S3, S5: "The numbers in bold indicate the highest performance obtained for a given structural level." - there aren't any numbers in bold.

*Response:We agree that the numbers in bold, used in Tables S3-S6 and Tables S9-S11, were not clearly visible. Therefore, we have changed the color of numbers in bold to blue to make them stand out from the surrounding text.*

***References:***

[*Altenhoff, Adrian M., Brigitte Boeckmann, Salvador Capella-Gutierrez, Daniel A. Dalquen, Todd DeLuca, Kristoffer Forslund, Jaime Huerta-Cepas, et al. 2016. “Standardized Benchmarking in the Quest for Orthologs.” Nature Methods 13 (5): 425–30.*](http://paperpile.com/b/jcDzMT/t78M)

[*Belmann, Peter, Johannes Dröge, Andreas Bremges, Alice C. McHardy, Alexander Sczyrba, and Michael D. Barton. 2015. “Bioboxes: Standardised Containers for Interchangeable Bioinformatics Software.” GigaScience 4 (October): 47.*](http://paperpile.com/b/jcDzMT/B7xT)

[*Bernard, Guillaume, Cheong Xin Chan, Yao-Ban Chan, Xin-Yi Chua, Yingnan Cong, James M. Hogan, Stefan R. Maetschke, and Mark A. Ragan. 2019. “Alignment-Free Inference of Hierarchical and Reticulate Phylogenomic Relationships.” Briefings in Bioinformatics 20 (2): 426–35.*](http://paperpile.com/b/jcDzMT/xTAh)

[*Bernard, Guillaume, Cheong Xin Chan, and Mark A. Ragan. 2016. “Alignment-Free Microbial Phylogenomics under Scenarios of Sequence Divergence, Genome Rearrangement and Lateral Genetic Transfer.” Scientific Reports 6 (July): 28970.*](http://paperpile.com/b/jcDzMT/1KQI)

[*Chan, Cheong Xin, and Mark A. Ragan. 2013. “Next-Generation Phylogenomics.” Biology Direct 8 (January): 3.*](http://paperpile.com/b/jcDzMT/oQAA)

[*Dewey, Colin N. 2012. “Whole-Genome Alignment.” Methods in Molecular Biology 855: 237–57.*](http://paperpile.com/b/jcDzMT/lcYt)

[*Earl, Dent, Ngan Nguyen, Glenn Hickey, Robert S. Harris, Stephen Fitzgerald, Kathryn Beal, Igor Seledtsov, et al. 2014. “Alignathon: A Competitive Assessment of Whole-Genome Alignment Methods.” Genome Research 24 (12): 2077–89.*](http://paperpile.com/b/jcDzMT/9Jhs)

[*Sage, Daniel, Hagai Kirshner, Thomas Pengo, Nico Stuurman, Junhong Min, Suliana Manley, and Michael Unser. 2015. “Quantitative Evaluation of Software Packages for Single-Molecule Localization Microscopy.” Nature Methods 12 (8): 717–24.*](http://paperpile.com/b/jcDzMT/xQEp)

[*Sczyrba, Alexander, Peter Hofmann, Peter Belmann, David Koslicki, Stefan Janssen, Johannes Dröge, Ivan Gregor, et al. 2017. “Critical Assessment of Metagenome Interpretation-a Benchmark of Metagenomics Software.” Nature Methods 14 (11): 1063–71.*](http://paperpile.com/b/jcDzMT/GsuQ)
